# Notes and News

**Published:** 1892-03-12

**Authors:** 


### Notes and News.

Collected and Communicated.

The twenty-fourth half-yearly report of Dr. Collingridge, the Medical Officer of Health for the Port of London, has just been issued. During the six months ending December 31st, 1891, 7,733 vessels of all classes underwent inspection by the sanitary officers; of those 6,516 or 84·26 per cent. carried the British flag. A proportion of 2·5 per cent. required cleansing, but this state of things shows a tendency to decrease, which is considered a satisfactory proof of the value of continuous sanitary supervision.

A responsible but difficult duty is that of the prevention of the "smoke nuisance," which under the new Act is thrown directly on the sanitary authority, without as hitherto the intervention of the police.

The report lays great stress on the necessity for some amendment of the law relating to cubic space, allowed to seamen of the Mercantile Marine. The present space, viz., 72 cubic feet, is considered too limited. It is shown that in many instances even this limited space is not always given, and also that it is not only the ordinary seaman who suffers in this respect, but his officers, who might be expected to enjoy some greater privileges with regard to personal accommodation. Nevertheless, some owners allow their crews considerably more than the required space, bnt this is stated not to apply as a rule to vessels recently built.

Particulars are given of a vessel having brought small-pox from Buenos Ayres, a severe confluent case, which was removed on arrival. Vaccination was offered to the crew, who refused. Some of them were subsequently paid off, and although undoubtedly infected, travelled through the country to Liverpool, Glasgow, and other places. By means of this crew, the disease was conveyed to Montreal, Glasgow, and Campbeltown, with in one instance fatal effect. The increasing tendency of ship masters to neglect to enter cases of sickness in their official logs having frequently come before the Port sanitary authority, the master of the s.s. "Clyde" was summoned and convicted in four instances. With regard to canal boats, the condition of the inhabitants is stated to be improving, and the working of the Acts to be popular to a greater extent than formerly amongst the class they are intended to protect.

With reference to the present mode of disposal of the metropolitan sewage, Dr. Collingridge, in his medical report for the Port of London, considers it absurd and unfair to criticise the working of a scheme which is not yet completed, and even when this is done, and the total diversion of all sewage matter from the river is accomplished, a considerable time must elapse before the river can attain its normal condition. At the present no improvement is perceptible.

Mr. Howard J. Collins has been appointed House Governor and Secretary to the Birmingham General Hospital out of 53 applicants. Mr. Collins has been Resident Secretary and House Steward of the Norfolk and Norwich Hospital for 3½ years. He was previously Secretary of the Hospitals Association for a year and a half, and Assistant Secretary and Accountant of the London Lock Hospital for nearly three years, so that he has had nearly eight years' practical experience of hospital work and management. We wish Mr. Collins all success in his new and important post.

Whilst the claims of shop women are coming to the fore, we take the opportunity of calling attention to an old grievance which is, in our opinion, much in need of reform, and one which it is strange should still remain unaltered. That is the absence of any means of rest for shop assistants when the opportunity of being seated occurs. That so many hours of uninterrupted standing can be healthy we decline to believe. In the case of nurses this has been admitted to be the case. Why philanthropic ladies have not made a determined crusade where so undoubted a grievance exists is to us a matter of surprise. Surely, were a few influential customers of some of the larger establishments to combine, and represent to the proprietors, in the cause of humanity their desire that seats should be provided for their shop assistants, the matter would be easily arranged, and the smaller shops would not be long in following the example of their betters. A small amount of combination is all that is necessary. Women have been accused of lack of power to combine. But they are learning to correct the errors of the past in this respect. In matters of philanthropy they have never been backward. Surely here is a cause worthy of some effort. Inventors have provided the market with ingeniously contrived seats, designed especially for counter use, only a little courage on the part of the powerful is needed to ensure their universal use.

The seventy-first annual meeting of the Seamen's Hospital Society was held last week at the Fishmongers' Hall, London Bridge. Mr. Charles H. Wilson, M.P., occupied the chair, and was supported by the Bishop of Colchester, Chevalier F. Krapf De Liverhoff (Austro-Hungarian Consul-General), Mr. E. A. Delcomyn (Consul-General for Denmark), Alderman Stuart Knill, Admiral H. D. Kantzow, Mr. G. Lidgett, Mr. H. C. Burdett, and others. The annual report states that in every branch of the society there has been increased activity, and the work had been carried on with much vigour. The number of patients treated at all the establishments of the society had been larger than in any year since the foundation of the society. At Greenwich alone there had been 2,642 in-patients, a larger number than in any year since the cholera epidemic of 1854. The branch hospital in the Royal Victoria and Albert Docks, which had now completed its first full year of active work, had been almost constantly full during the year. The expenses of the society have exceeded the income by £2,479 15s. 8d. The ordinary income, exclusive of legacies, showed an increase over last year's ordinary receipts of about £607 17s. 9d., which sum, although encouraging, is not sufficient to meet the extra expenditure entailed by the greatly increased work done by the society. It is proposed to establish a branch hospital in the neighbourhood of Tilbury Docks, and an offer had been made of a suitable building for the purposes of a hospital, entirely free of cost, together with a sum of £396 towards the preliminary expenses. The work of the Samaritan fund continued to be one of the most important features of the society.

